# Therapeutic significance of targeting survivin in cervical cancer and possibility of combination therapy with TRAIL

**DOI:** 10.18632/oncotarget.24413

**Published:** 2018-02-05

**Authors:** Hiroe Nakamura, Ayumi Taguchi, Kei Kawana, Satoshi Baba, Akira Kawata, Mitsuyo Yoshida, Asaha Fujimoto, Juri Ogishima, Masakazu Sato, Tomoko Inoue, Haruka Nishida, Hitomi Furuya, Aki Yamashita, Satoko Eguchi, Kensuke Tomio, Mayuyo Mori-Uchino, Katsuyuki Adachi, Takahide Arimoto, Osamu Wada-Hiraike, Katsutoshi Oda, Takeshi Nagamatsu, Yutaka Osuga, Tomoyuki Fujii

**Affiliations:** ^1^ Department of Obstetrics and Gynecology, Graduate School of Medicine, The University of Tokyo, Bunkyo-ku, Tokyo 113-8655, Japan; ^2^ Department of Obstetrics and Gynecology, Nihon University School of Medicine, Itabashiku, Tokyo 173-8610, Japan

**Keywords:** survivin, cervical cancer, TRAIL, human papilloma virus, resveratrol

## Abstract

Loss of p53 function due to human papillomavirus (HPV) infection induces resistance to apoptosis in cervical cancer cells. Tumor necrosis factor-related apoptosis-inducing ligand (TRAIL), which induces apoptosis in a p53-independent manner, may provide an alternative strategy for treating cervical cancer. Survivin, an antiapoptotic protein that is highly expressed in cancer cells, regulates apoptosis and the cell cycle. Here, we investigated the therapeutic potential of targeting survivin, while focusing on the TRAIL-induced apoptosis pathway. The viability and cell cycle of HPV16-positive CaSki and SiHa cells were assessed after survivin knockdown by small interfering RNA (si-survivin). E-cadherin expression was also assessed after si-survivin treatment, using western blotting. SiHa (a TRAIL-resistant cell line) was used for further studies. The small molecule YM155 and resveratrol (RVT; a polyphenol with the potential to suppress survivin expression) were used as survivin inhibitors. The effects of si-survivin and survivin inhibitors on TRAIL- or cisplatin (CDDP)-induced apoptosis were analyzed by annexin-V staining. si-survivin treatment decreased cell viability and led to G2/M arrest, accompanied by morphological changes and E-cadherin upregulation in both CaSki and SiHa cells. si-survivin and YM155 synergistically sensitized TRAIL-resistant SiHa cells to TRAIL-induced apoptosis (*p* < 0.05). However, si-survivin and YM155 only slightly increased CDDP-induced apoptosis. RVT markedly enhanced TRAIL-induced apoptosis by suppressing survivin expression. Targeting of survivin expression might be an ideal strategy for cervical cancer treatment as it would decrease viable cell number and enhance apoptosis sensitivity. Further, combination therapy with TRAIL, rather than CDDP, may be compatible with the proposed survivin-targeting strategy.

## INTRODUCTION

Cervical cancer is the third most commonly diagnosed cancer and the fourth leading cause of cancer death globally among female individuals [1]. For recurrent or locally advanced cervical cancer, cisplatin (CDDP)-based chemotherapy is reported to be the most effective treatment; however, the response rate is not sufficient, ranging from 20% to 50%, and the expected overall survival is only 10 to 17.5 months [2, 3].

Human papillomavirus (HPV) infection is responsible for most cases of invasive cervical cancer [4]. The HPV oncoproteins E6 and E7 suppress the function of the tumor suppressor genes p53 and the retinoblastoma gene product pRb, respectively. The HPV E6 gene product binds to p53 and targets it for rapid degradation via a cellular ubiquitin ligase [5]. As a consequence, the essential function of p53, which controls cell cycle, apoptosis, and DNA repair, is abrogated [5, 6]. Considering the low response rate to chemotherapy and the unique characteristics of p53-abrogated cervical cancer, strategies to enhance the response to CDDP and development of other types of combination therapies are urgently required.

Tumor necrosis factor-related apoptosis-inducing ligand (TRAIL) is a member of the TNF superfamily. TRAIL induces apoptosis in a p53-independent manner, and it has strong antitumor activity with minimal cytotoxicity to normal cells [7–9]. Recombinant TRAIL (dulanermin) and TRAIL-receptor agonists (mapatumumab, drozitumab, and conatumumab) have already been tested in some clinical trials [10]. It therefore shows great promise for treatment of cervical cancer.

Hougardy *et al.* demonstrated that the HPV16-positive cervical cancer cell line SiHa is resistant to TRAIL-induced apoptosis, whereas the HPV16-positive line CaSki is sensitive [11].

As a strategy for treatment of cervical cancer, we previously proposed combination therapy with the STAT3 inhibitor S3I-201 and tumor necrosis factor-related apoptosis-inducing ligand (TRAIL), and tested this strategy using SiHa cells [12]. Decreased STAT3 activation results in sensitization to TRAIL-induced apoptosis, even in the TRAIL-resistant cervical cancer cell line SiHa [12]. However, given the normal role of STAT3 in cell proliferation, survival, development, and differentiation [13, 14], inhibition of STAT3 activation might disrupt normal biological responses.

In this context, we focused on survivin, an important antiapoptotic molecule that is usually overexpressed only in malignant cells [15]. Survivin, a member of the inhibitor of apoptosis (IAP) gene family, is known to regulate apoptosis and the cell cycle [16, 17]. Survivin is expressed in the embryonic lungs and fetal organs during development but is undetectable in most normal adult tissues [17, 18]. Compared to terminally differentiated tissue, most cancer cells express survivin at higher levels [18, 19]. Therefore, survivin is considered an ideal target for cancer therapy. The survivin inhibitor YM155 (sepantronium bromide) is a small imidazolium-based compound (1-(2-methoxyethyl)-2-methyl-4,9-dioxo-3-(pyrazin-2-ylmethyl)-4,9-dihydro-1*H*-naphthimidazolium bromide) whose mechanism of action involves a 2-kb promoter region of the survivin gene [20]. It has been tested in some clinical trials [20, 21] and is an attractive option as a molecular targeting agent in a clinical setting.

Numerous studies have reported that increased survivin expression levels are correlated with poor prognosis in cervical cancer [22, 23]. Kogo *et al.* demonstrated that microRNA218 contributed to more aggressive tumor formation via survivin overexpression and that survivin knockdown reduced the invasive ability of cervical cancer cells [24]. Given these findings, survivin is expected to be an ideal target for cervical cancer treatment. Moreover, survivin has been reported to contribute to TRAIL resistance, and survivin suppression has been found to enhance TRAIL-induced apoptosis [25, 26].

Although the contribution of survivin to the modulation of invasiveness has been well demonstrated in cervical cancer cells [24], whether survivin can serve as a therapeutic target for the purpose of inducing apoptosis remains unknown. In this study, we demonstrated the therapeutic potential of targeting survivin, focusing on the induction of apoptosis.

## RESULTS

### Survivin knockdown induced G2/M arrest accompanied by morphological changes and E-cadherin upregulation in cervical cancer cell lines

**Figure 1:**
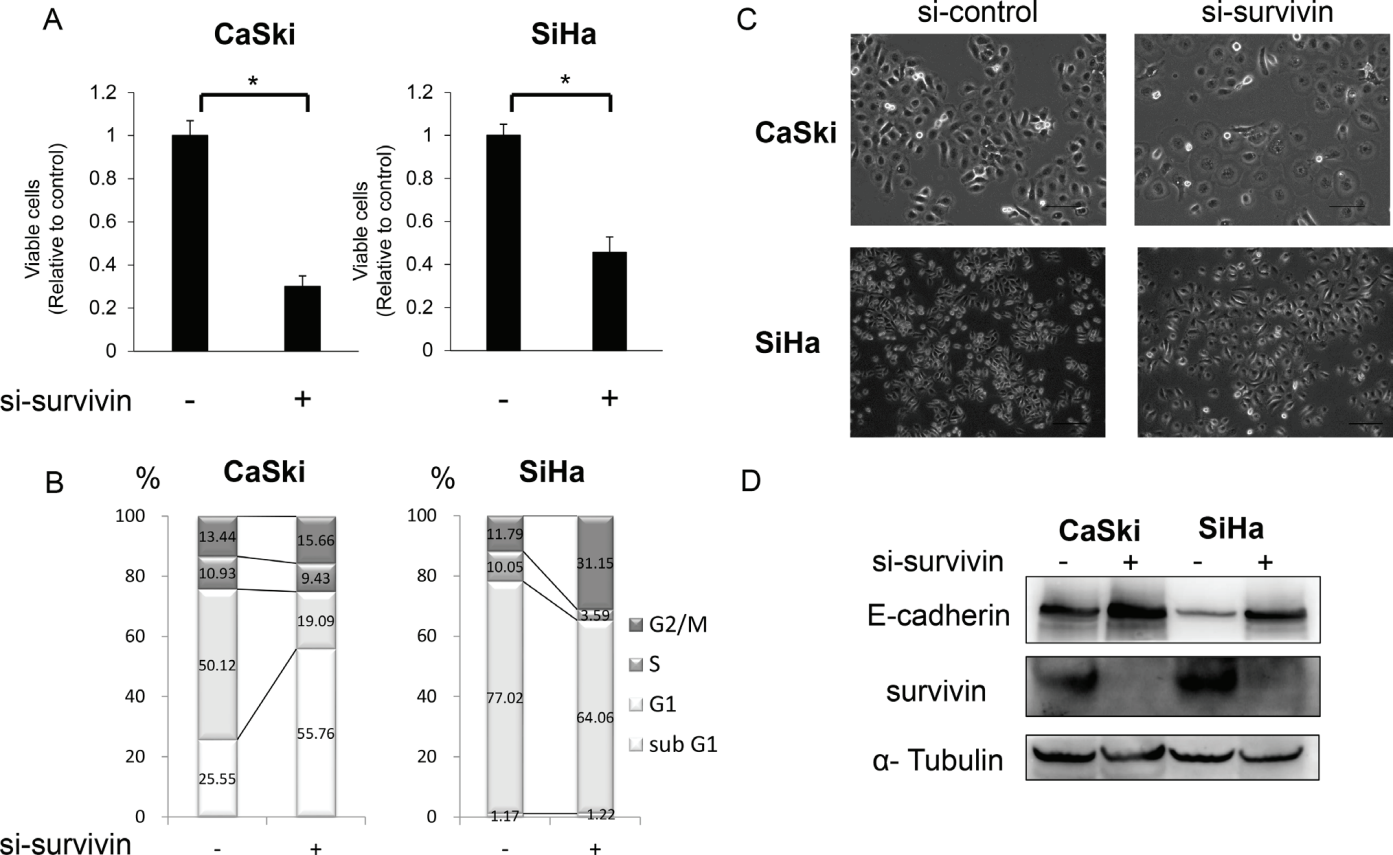
Effects of survivin suppression on viability, cell cycle, and E-cadherin expression in cervical cancer cell lines (**A**) Viability of CaSki and SiHa cells after survivin knockdown. CaSki and SiHa cells were transfected with survivin-specific siRNA (si-survivin) for 48 h and then adherent cells were counted to assess their viability. The experiment was performed in triplicate. The cell numbers were normalized relative to control cells. Data are provided as mean (±SEM) values. The data were analyzed using Student’s *t*-test.^*^P < 0.05. (**B**) Flow cytometric analysis of the cell cycle after survivin knockdown. CaSki and SiHa cells were transfected with survivin-specific siRNA (si-survivin) for 48 h and then the cell cycle was analyzed. The mean of three independent experiments is shown. (**C**) Effects of survivin knockdown on the morphology of CaSki and SiHa cells. CaSki and SiHa cells were transfected with survivin-specific siRNA (si-survivin) for 48 h and then the image was captured using a fluorescence microscope. Scale bars indicate 100 μm. (**D**) E-cadherin expression after survivin knockdown. CaSki and SiHa cells were transfected with survivin-specific siRNA (si-survivin) for 48 h and lysed in cell lysis buffer. Then, E-cadherin expression was analyzed by western blotting.

Because survivin has been reported to control mitosis and the cell cycle, as well as cell proliferation [18], we first investigated the effect of survivin on cell viability by determining cell counts after survivin knockdown. Survivin knockdown (si-survivin) significantly decreased the number of viable cells in both CaSki and SiHa cells (CaSki:0.30 [±0.08] fold (*p* = 0.001), SiHa: 0.46 [±0.12] fold (*p* = 0.002); Figure 1A). Then, we investigated the effect of survivin knockdown on the cell cycle. Knockdown of survivin expression resulted in G2/M arrest in both cell lines (Figure 1B). Survival of CaSki cells was dependent on survivin expression to a greater extent than survival of SiHa cells; survivin downregulation led to an increased number of sub-G1 populations, indicating that it increased the number of apoptotic cells (Figure 1B). We also investigated the effect in the HeLa cell line, an HPV18-positive cervical cell line, to confirm that the effect was not specific to HPV16-positive cervical cancer cell lines such as SiHa and CaSki. Survivin suppression decreased the number of viable cells and induced G2/M arrest in HeLa cells as well (Supplementary Figure 1).

Kogo *et al.* previously demonstrated that survivin is responsible for the invasive ability of cervical cancer cells [24]. Therefore, we investigated morphological changes, as well as modulation of E-cadherin, an epithelial–mesenchymal transition (EMT) marker, following knockdown of survivin expression. E-cadherin was upregulated by survivin inhibition, accompanied by morphological changes in both cell lines (Figure 1C and 1D).

### Survivin knockdown and TRAIL combination therapy decreased viable cell number

**Figure 2:**
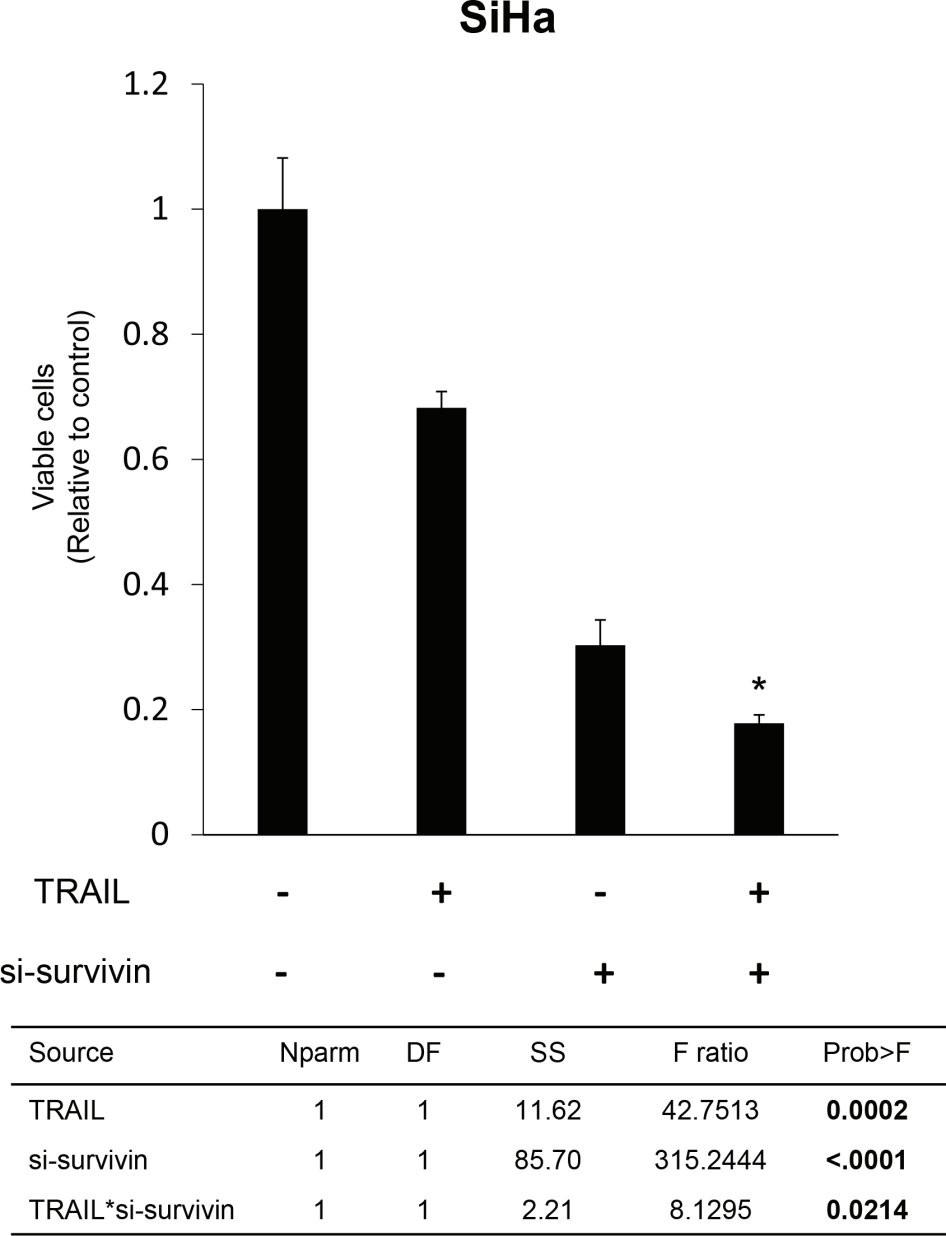
Effect of survivin suppression and TRAIL combination therapy on cell viability in TRAIL-resistant SiHa cells SiHa cells were transfected with survivin-specific siRNA (si-survivin) for 48 h and then treated with TRAIL (100 ng/mL) for an additional 24 h. Adherent cells were counted to assess cell viability. The experiment was performed in triplicate. Cell numbers were normalized relative to control cells. Data are provided as mean (±SEM) values. Two-way ANOVA results are provided below. Asterisk (^*^) indicates that the *p*-value of the interaction effect was < 0.05. Nparm: number of parameters, DF: degree of freedom, SS: sum of squares.

We investigated whether knockdown of survivin expression and TRAIL combination therapy affected viable cell number. Survivin knockdown and TRAIL combination therapy synergistically suppressed viable cell numbers, more effectively than TRAIL monotherapy (the *p*-value of interaction effect evaluated by two-way ANOVA was 0.0214) (Figure 2).

### si-survivin and the survivin-specific inhibitor YM155 synergistically enhanced sensitivity to TRAIL-induced apoptosis

**Figure 3:**
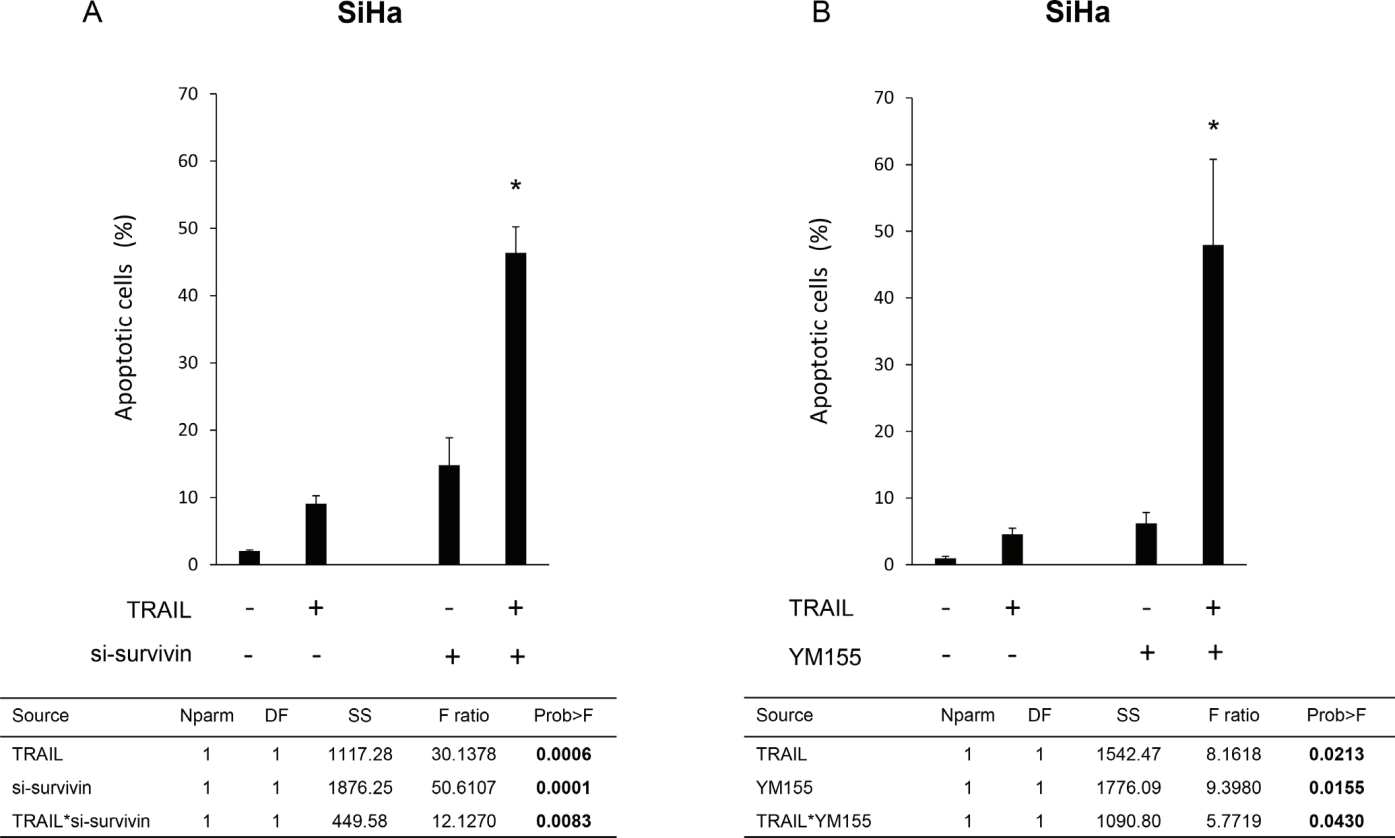
Effects of survivin suppression on TRAIL-induced apoptosis in TRAIL-resistant SiHa cells (**A**) Effects of survivin knockdown on TRAIL-induced apoptosis in SiHa cells. SiHa cells were transfected with control siRNA or survivin-specific siRNA for 48 h, and then treated with TRAIL (100 ng/mL) or not treated for an additional 15 h. The proportion of apoptotic cells was evaluated using annexin-V–fluorescein isothiocyanate (FITC) and propidium iodide (PI) double staining, followed by flow cytometry. The results show the mean of three independent experiments (± SEM). Two-way ANOVA results are provided below in the column. Asterisks (*) indicate that the *p*-value of the interaction effect was < 0.05. (**B**) Effect of the survivin inhibitor YM155 on TRAIL-induced apoptosis in SiHa cells. SiHa cells were treated with YM155 (20 nM) for 24 h, and then treated with TRAIL (100 ng/mL) or not treated for an additional 15 h. The proportion of apoptotic cells was evaluated as described in Figure 3A. Two-way ANOVA results are provided below. Asterisks (*) indicate that the *p*-value of the interaction effect was < 0.05.

We investigated whether knockdown of survivin expression affected TRAIL-induced apoptosis in TRAIL-resistant SiHa cells. We found that TRAIL induced apoptosis in only 9.10 [±1.17]% of SiHa cells (Figure 3A). However, knockdown of survivin expression markedly boosted TRAIL-induced apoptosis in SiHa cells. (si-survivin alone 14.8 [±4.07]%, si-survivin + TRAIL 46.34 [±3.88]%.) The two-way ANOVA demonstrated that the *p*-value of interaction effect of combination therapy was 0.0083, indicationg that the effect was synergistic, rather than additive. We also tested another siRNA sequence against survivin and confirmed that the result was consistent with the results shown in Figure 3A (Supplementary Figure 2).

We then investigated the effect of the survivin inhibitor YM155 on TRAIL-induced apoptosis. We found that 20 nM YM155 also sensitized SiHa cells to TRAIL-induced apoptosis (TRAIL alone 4.56 [±0.91]%, YM155 alone 6.21 [±1.06]%, YM155 + TRAIL 48.0 [±12.8]%; the *p*-value of interaction effect evaluated by two-way ANOVA was 0.043; Figure 3B).

We next invtigated the effect of survivin knockdown on TRAIL-induced apoptosis in HeLa cells, which are reported to be moderately sensitive to TRAIL [11]. Approximately half of the cells (57.5 [±4.78]%) underwent apoptosis following treatment with 100 ng/ml of TRAIL. Si-survivin and TRAIL combination therapy additively induced apoptosis in as many as 87.5 [±1.60]% of cells (Supplementary Figure 3). The two-way ANOVA analysis demonstrated that the effect was not synergistic but rather additive (the *p*-value of interaction effect evaluated by two-way ANOVA was 0.2396).

### si-survivin and the survivin-specific inhibitor YM155 did not enhance CDDP-induced apoptosis

**Figure 4:**
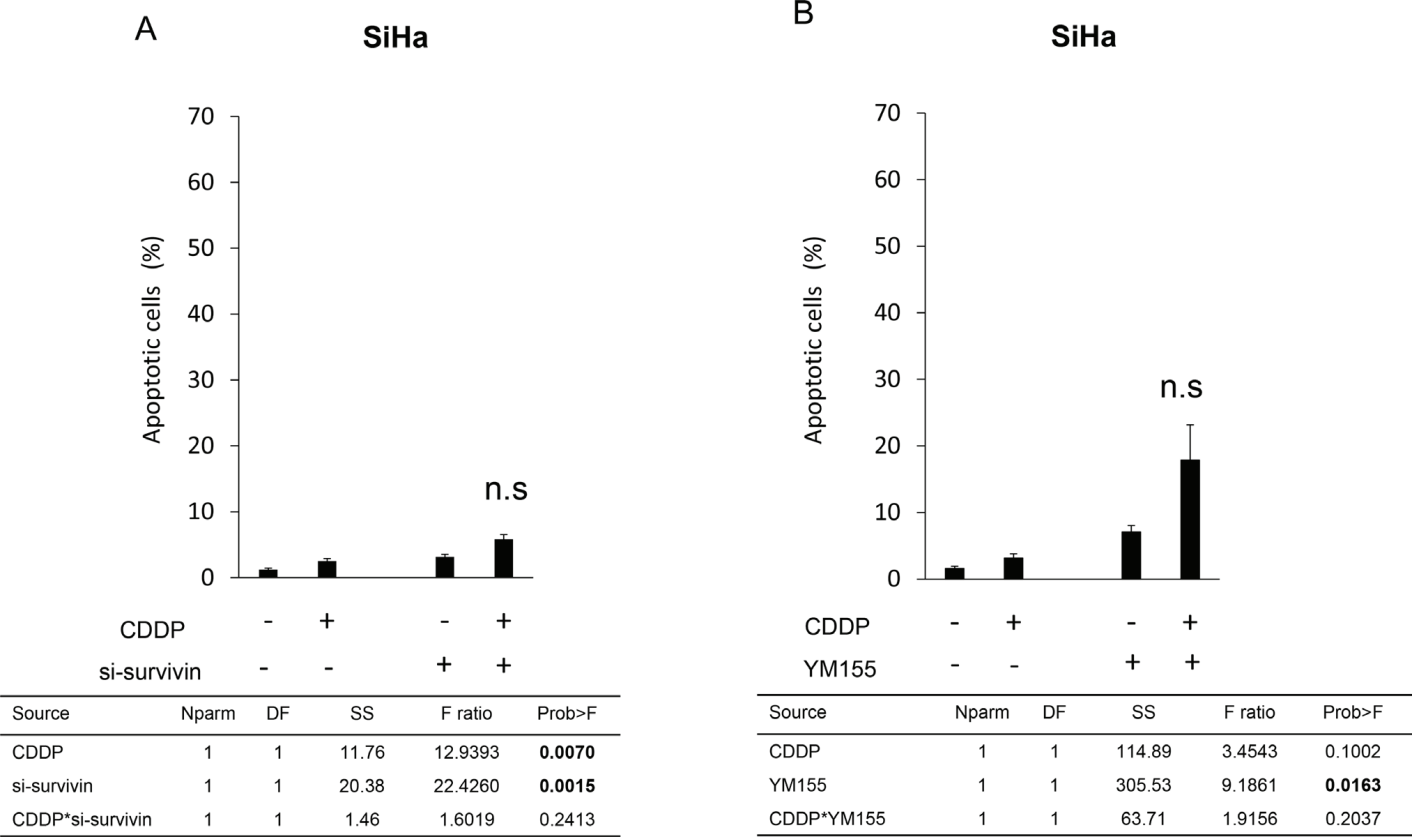
Effects of survivin suppression on CDDP-induced apoptosis in SiHa cells (**A**) SiHa cells were transfected with control siRNA or survivin-specific siRNA for 48 h and then treated with CDDP (20 μM) or not treated for an additional 24 h. The proportion of apoptotic cells was evaluated as described in Figure 3A. The results show the mean of three independent experiments (±SEM). The data were analyzed using two-way ANOVA. n.s.: the interaction effect was not significant. (**B**) SiHa cells were treated with YM155 (20 nM) for 24 h and then treated with CDDP (20 μM) or not treated for an additional 24 h. The proportion of apoptotic cells was evaluated as described in Figure 3A. The results show the mean of three independent experiments (±SEM). The data were analyzed using two-way ANOVA. n.s.: the interaction effect was not significant.

We next investigated the effect of survivin on CDDP-induced apoptosis in SiHa cells. Knockdown of survivin with small interfering RNA (siRNA) only slightly increased CDDP-induced apoptosis in SiHa cells; the effect was not synergistic (CDDP alone 2.53 [±0.36]%, si-survivin alone 3.15 [±0.37]%, si-survivin + CDDP 5.83 [±0.71]%; the *p*-value of interaction effect evaluated by two-way ANOVA was 0.2413; Figure 4A). We also assessed YM155 in relation to CDDP-induced apoptosis and found that its effects were not sufficient to enhance CDDP-induced apoptosis in SiHa cells (CDDP alone 3.16 [±0.62]%, YM155 alone 7.06 [±1.00]%, YM155 + CDDP 17.9 [±5.30]%; the *p*-value of interaction effect evaluated by two-way ANOVA was 0.2037; Figure 4B).

As CDDP is known to suppress cell viability [27], we also investigated the effect of survivin suppression and CDDP combination therapy by analyzing cell viability. However, it did not produce a synergistic effect (Supplementary Figure 4).

### RVT suppressed survivin expression

**Figure 5:**
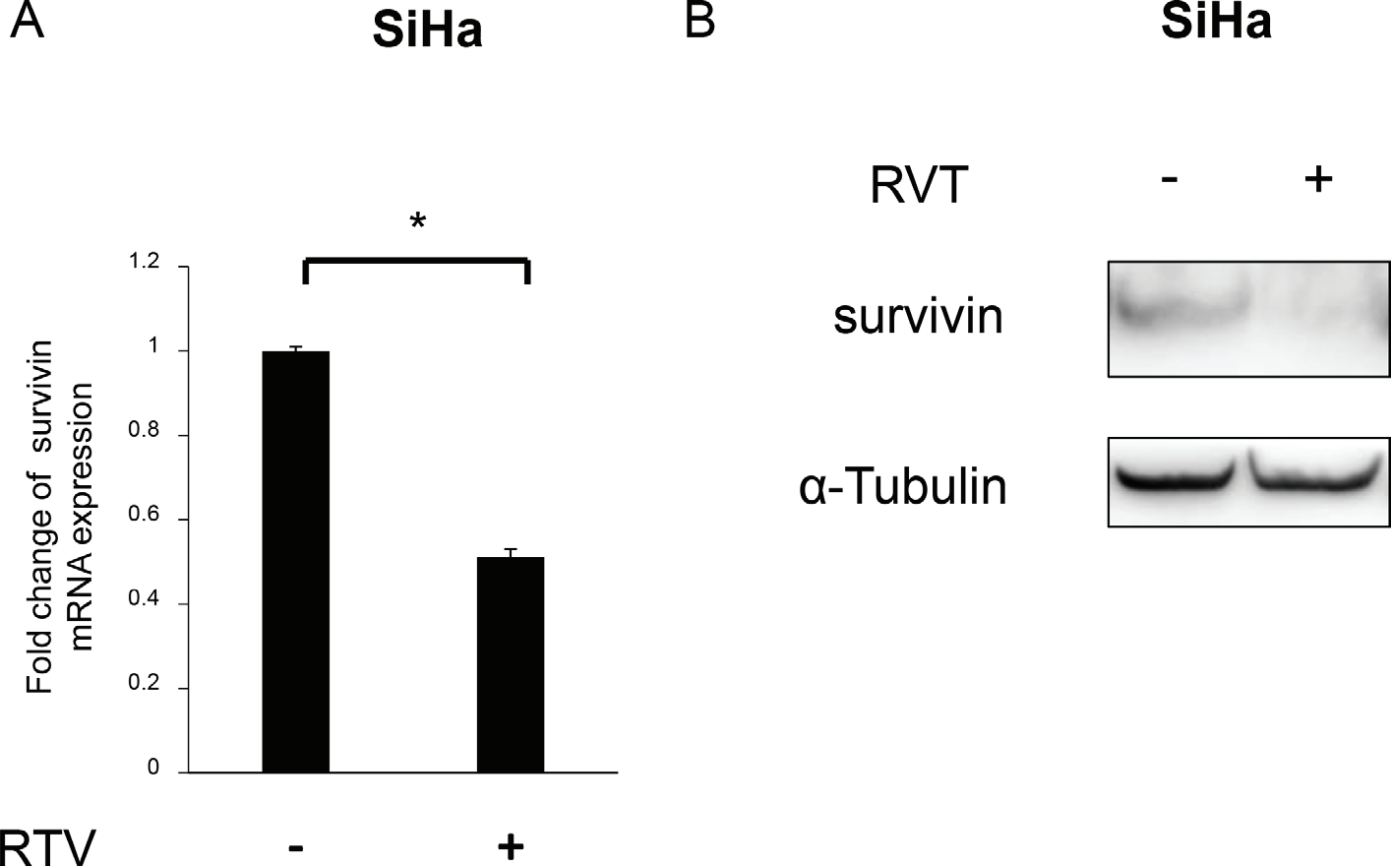
Effects of resveratrol (RVT) on survivin expression in SiHa cells (**A**) SiHa cells were treated with RVT (100 μM) for 24 h. Total RNA was reverse transcribed and the mRNA levels of survivin were measured via quantitative reverse transcription polymerase chain reaction. The expression level was normalized relative to that of glyceraldehyde-3-phosphate dehydrogenase (GAPDH). The mean (±SEM) values of three independent experiments are shown, and data were analyzed by Student’s *t*-test. ^*^P < 0.05. (**B**) SiHa cells were treated with RVT (100 μM) for 24 h and then lysed in cell lysis buffer. Subsequently, survivin expression was analyzed by western blotting.

RVT, a phytoalexin polyphenol produced naturally by several plants, is known to inhibit survivin expression, as well as STAT3 activation [28]. Zhang *et al.* previously reported that RVT inhibited STAT3 signaling in cervical cancer cell lines, including SiHa cells [29]. We confirmed that RVT inhibited STAT3 activation in SiHa cells (data not shown). We previously demonstrated that RVT enhanced TRAIL-induced apoptosis in endometriotic cells by suppressing survivin expression [30]. We then hypothesized that, in addition to STAT3 inhibition, RVT could enhance TRAIL-induced apoptosis in SiHa cells by suppressing survivin expression. We investigated whether RVT suppressed survivin expression in SiHa cells. RVT suppressed survivin expression in SiHa cells at both mRNA and protein levels (Figure 5A and 5B).

### RVT enhanced TRAIL-induced apoptosis in TRAIL-resistant SiHa cells

**Figure 6:**
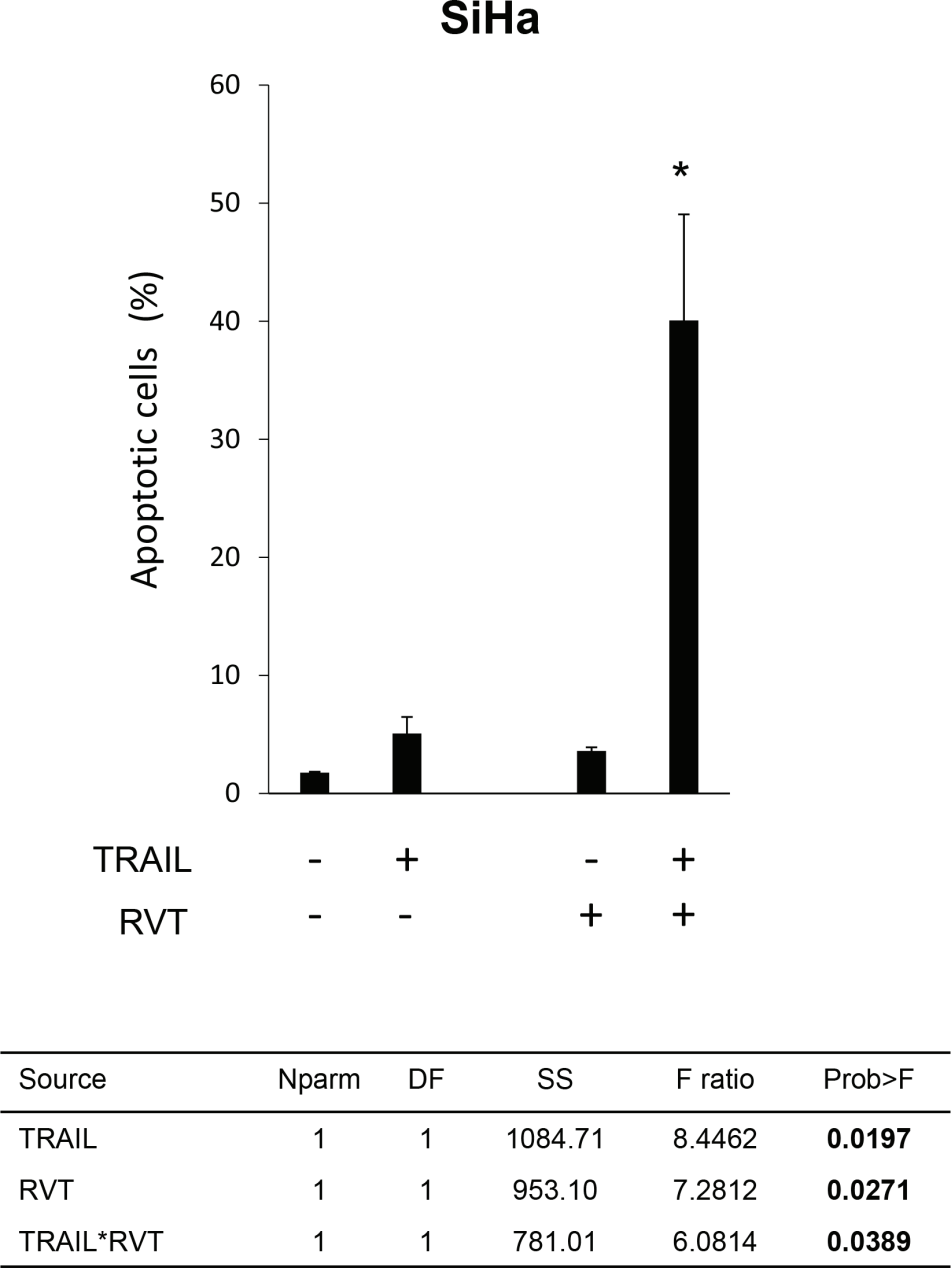
Effects of resveratrol (RVT) on TRAIL-induced apoptosis in SiHa cells SiHa cells were treated with RVT (100 μM) for 24 h, and then treated with TRAIL (100 ng/mL) or not treated for an additional 15 h. The proportion of apoptotic cells was evaluated using annexin-V–fluorescein isothiocyanate (FITC) and propidium iodide (PI) double staining, followed by flow cytometry. The results show the mean of three independent experiments (± SEM). Two-way ANOVA results are provided below. Asterisks (*) indicate that the *p*-value of the interaction effect was < 0.05.

Because RVT effectively suppressed survivin expression and STAT3 activity in SiHa cells, we next investigated the effects of RVT on TRAIL-induced apoptosis in SiHa cells. RVT alone barely induced apoptosis in SiHa cells, although pretreatment with RVT synergistically enhanced TRAIL-induced apoptosis (TRAIL alone 5.08 [±1.41]%, RVT alone 3.60 [±0.30]%, RVT + TRAIL 40.1 [±8.98]%; the *p*-value of interaction effect evaluated by two-way ANOVA was 0.0389; Figure 6).

## DISCUSSION

We analyzed the therapeutic potential of targeting survivin, focusing on the TRAIL-induced apoptosis pathway. We found that survivin downregulation led to G2/M arrest in the HPV16-positive cervical cancer cell lines CaSki and SiHa. Survivin downregulation also led to morphological changes accompanied by E-cadherin upregulation. Survivin knockdown and survivin inhibition enhanced TRAIL-induced apoptosis in TRAIL-resistant SiHa cells (Figure 3). However, survivin knockdown or inhibition only additively enhanced CDDP-induced apoptosis in SiHa cells (Figure 4). RVT suppressed survivin expression and enhanced TRAIL-induced apoptosis in SiHa cells (Figures 5 and 6).

In the current study, survivin knockdown with siRNA led to G2/M arrest in CaSki and SiHa cells. At the end of the S phase, the activated cyclin B/CDK1 complex triggers entry into mitosis [31]. Survivin is induced by this activated complex and contributes to appropriate creation of the mitotic spindle [32]; its expression reaches a peak in the G2/M phase [18]. Abnormal formation of the mitotic spindle by knockdown of survivin expression may have caused cell cycle arrest in the G2/M phase in the CaSki and SiHa cells, which is consistent with the results of other studies [33, 34].

Modulation of the invasive ability of cervical cancer cells by survivin was demonstrated by Kogo *et al;* the invasive ability was found to decrease on survivin suppression [24]. EMT, characterized by loss of cell polarity and morphological alterations, is a key step in cancer invasion [35]. Therefore, we focused on the effect of survivin knockdown on E-cadherin, a primary EMT marker. Survivin knockdown with siRNA led to upregulation of E-cadherin, accompanied by morphological changes (Figure 1C and 1D). This result indicates that survivin may control the invasive ability of cervical cancer cell lines through modulation of E-cadherin expression.

Survivin is a central regulator of cell apoptosis. Therefore, we investigated the effects of survivin suppression on the apoptosis of cervical cancer cells. Knockdown of survivin itself did not induce apoptosis of SiHa cells. Several reports have demonstrated that, although knockdown of survivin itself does not induce apoptosis, it influences the sensitivity to additional apoptotic stimuli [30]. Here, TRAIL and CDDP were chosen as the additional apoptotic stimuli. Among cervical cancer cell lines, SiHa has been reported to show the greatest resistance to TRAIL-induced apoptosis [11]; it is also resistant to CDDP [36]. Downregulation of survivin using siRNA or the survivin inhibitor YM155 synergistically enhanced the sensitivity to TRAIL-induced apoptosis and only slightly enhanced CDDP-induced apoptosis (Figures 3 and 4).

Differences in the mechanisms by which each apoptotic stimulus intracellularly induces apoptosis might be responsible for the difference in the contribution of survivin to TRAIL- and CDDP-induced apoptosis. Subsequent activation of the caspase-8 pathway is indispensable for TRAIL-induced apoptosis; therefore, the balance between proapoptotic signaling and antiapoptotic signaling determines cell death fate [37]. In CDDP-induced apoptosis, abnormal DNA structure induces DNA damage–dependent apoptosis [38]. In this case, enhancement of DNA damage sensors would be much more effective than suppression of antiapoptotic molecules for boosting apoptosis [39].

In the current study, RVT also suppressed survivin expression in SiHa cells (Figure 5A and 5B). RVT has been reported to be a potent inhibitor of STAT3 activation [28, 40]. STAT3 is known to be a transcription factor for survivin [14]. We previously reported that suppression of phosphorylated STAT3 (pSTAT3) also enhanced TRAIL-induced apoptosis in SiHa cells [12]. In our experiment, RVT also suppressed STAT3 activation (data not shown). Taken together, these co-suppressive effects of RVT might cooperatively enhance TRAIL-induced apoptosis in SiHa cells (Figure 6).

In contrast to TRAIL-induced apoptosis, RVT did not influence CDDP-induced apoptosis in CaSki or SiHa cells (data not shown). As discussed earlier, this difference between TRAIL- and CDDP-induced apoptosis might be caused by differences in the mechanism underlying apoptosis.

In conclusion, we propose that targeting of survivin expression, to decrease the viable cell number and enhance sensitivity to apoptosis, might be an ideal strategy for cervical cancer treatment. TRAIL-based combination therapy may be compatible with strategies for directly or indirectly targeting survivin.

## MATERIALS AND METHODS

### Antibodies and reagents

For western blotting, the following antibodies were used at the dilution indicated: mouse anti-alpha tubulin sc-8035 (1:500), rabbit anti-survivin (CS#2808; 1:1000; Cell Signaling Technologies, Massachusetts, USA), and mouse anti-E-cadherin (BD610181; 1:500, BD, California, USA). YM155 was purchased from Merck Millipore (Darmstadt, Germany), resveratrol (RVT) from Sigma Aldrich (Montana, USA), and recombinant human TRAIL from R&D Systems (Minnesota, USA).

### Cell culture

The HPV16-positive cervical cancer cell lines CaSki and SiHa (purchased from ATCC, Virginia, USA) were maintained in Dulbecco’s Modified Eagle Medium supplemented with 10% FBS (Life Technologies, California, USA) and antibiotics (Antibiotic-Antimycotic Mixed Stock Solution; Nacalai Tesque, Kyoto Japan). The cells were grown in a humidified tissue culture incubator at 37° C in 5% CO_2_.

### Cell proliferation assay

To analyze the effect of survivin knockdown on cell proliferation, adherent viable cells were counted by trypsinization. Cell counting was performed by counting the number of cells in 1 mL of collected medium by using trypan blue staining. Cell number was normalized relative to the number of control cells.

### Cell cycle analysis

Cell cycle analysis was performed as previously described [41]. Cells were seeded in 10-cm dishes and transfected with survivin-specific siRNA for 48 h. Floating and adherent cells were collected by trypsinization and washed twice with PBS.

Cells were resuspended in cold 70% ethanol and maintained at 4° C overnight. After they were washed twice with PBS, they were incubated in RNase A (0.25 mg/mL; Sigma Aldrich) for 30 min at 37° C, followed by staining with propidium iodide (PI; 50 μg/mL; Sigma Aldrich) at 4° C for 30 min in the dark. The cells were then analyzed using flow cytometry (BD FACSCalibur HG, New Jersey, USA). The cell cycle distribution was analyzed using Cell Quest Pro ver. 3.1. (Beckman Coulter Epics XL, California, USA). Three independent experiments were performed.

### Detection of apoptosis by staining with annexin-V FITC

Cells (4 × 10^5^/well) were cultured in 60-well plates for 24 h before treatment. Then, they were transfected with siRNA for 48 h or YM155 for 24 h, with an additional 15–18 h of TRAIL treatment. The cells were trypsinized, washed with PBS, and then analyzed after double staining with the Annexin-V Apoptosis Detection Kit (Abcam, Massachusetts, USA). The apoptotic cell population was analyzed using flow cytometry. All experiments were performed three times.

### Immunoblotting

Immunoblotting was performed as previously described [12]. Cells were lysed by incubation in lysis buffer (Cell Signaling Technologies) containing a protease-inhibitor cocktail (Nacalai Tesque) and a phosphatase-inhibitor cocktail (Roche, Mannheim, Germany) on ice for 5 min and sonicated briefly. Then, they were centrifuged at 14,000 rpm at 4° C for 10 min, and the supernatant was used for analysis. For SDS-PAGE, 20 μg of protein lysate was loaded in each well. For immunoblotting, 0.45 μm polyvinylidene difluoride (PVDF) membranes (Merck Millipore) were used. The membranes were blocked in 5% milk/TBS-T (TBS containing 0.1% Tween-20) for 1 h at 22–26° C followed by incubation with the primary antibodies diluted in 5% milk/TBS-T or 5% bovine serum albumin (BSA)/TBS-T for an appropriate duration according to the manufacturer’s instructions. After the membranes were washed several times with TBS-T, they were incubated with secondary antibodies conjugated with horseradish peroxidase (HRP) in 5% milk/TBS-T at 22–26° C for 1 h. The blots were developed using Immobilon Western Chemiluminescent HRP substrate (Merck Millipore) according to the manufacturer’s instructions.

### Transfection

siRNA transfections were performed using Stealth RNAi against survivin (BIRC5) (HSS179403, HSS179404) and non-targeting siRNA (Stealth RNAi siRNA Negative Control; Med GC, Life Technologies) as a control. When 60–70% confluency was achieved, the transfections were performed using Lipofectamine RNAiMAX (Life Technologies), Opti-MEM Reduced Serum Medium (Life Technologies), and siRNAs (final concentration, 20 nmol/L), according to the manufacturer’s instructions. After 5 h of incubation, the transfection medium was changed to normal culture medium without antibiotics. The cells were incubated for 48 h and then analyzed for each experiment. The transfection sequence was repeated at least three times.

### RT-qPCR

Total RNA was extracted from cells using the Blood/Cultured Cell Total RNA Mini Kit (FAVORGEN, Ping Tung, Taiwan), followed by reverse transcription using ReverTra Ace qPCR RT Master Mix (Toyobo, Osaka, Japan) according to the manufacturer’s instructions. cDNA was amplified for 40 cycles in a Light Cycler 480 (Roche) using LightCycler 480 SYBR Green I Master reagent (Roche). Expression of survivin was normalized to that of GAPDH mRNA (internal standard) by the ΔΔCt method. The primer pairs were as follows (final concentration 0.5 μM): human survivin, 5′-GGACCACCGCATCTCTACAT-3′ and 5′-GC ACTTTCTTCGCAGTTTCC-3′; human GAPDH, 5′-GAAA GGTGAAGGTCGGAGTC-3′ and 5′-GAAGATGGTGATG GGATTTC-3′. Three independent experiments were performed.

### Cell imaging

The effect of survivin knockdown was assessed using a fluorescence microscope (BZ-9000; Keyence, Osaka, Japan).

### Statistical analysis

Data are presented as the mean ± standard error of the mean (SEM). Statistical analyses were performed using Student’s *t*-test with JMP software (SAS, North Carolina, USA). *P* < 0.05 was considered significant. Two-way factorial analysis of variance tests (two-way ANOVA) were performed with JMP software to assess whether combination therapy produced an additive effect or a synergistic effect. The *p*-value of interaction effect is demonstrated at the last line of each column in each figure (Figures 2–4, 6 and Supplementary Figures 2,3) When the *p*-value of the interaction effect was < 0.05, the effect was considered synergistic.

## SUPPLEMENTARY MATERIALS FIGURES


