# Supplementary material for: Therapeutic significance of targeting survivin in cervical cancer and possibility of combination therapy with TRAIL
Source: Oncotarget. 2018 Feb 5;9(17):13451–61. doi: 10.18632/oncotarget.24413 (PMC5862590; doi:10.18632/oncotarget.24413)
Supplement: Supplementary file 1 [file oncotarget-09-13451-s001.pdf]

Therapeutic significance of targeting survivin in cervical cancer and possibility of combination therapy with TRAIL

SUPPLEMENTARY MATERIALS

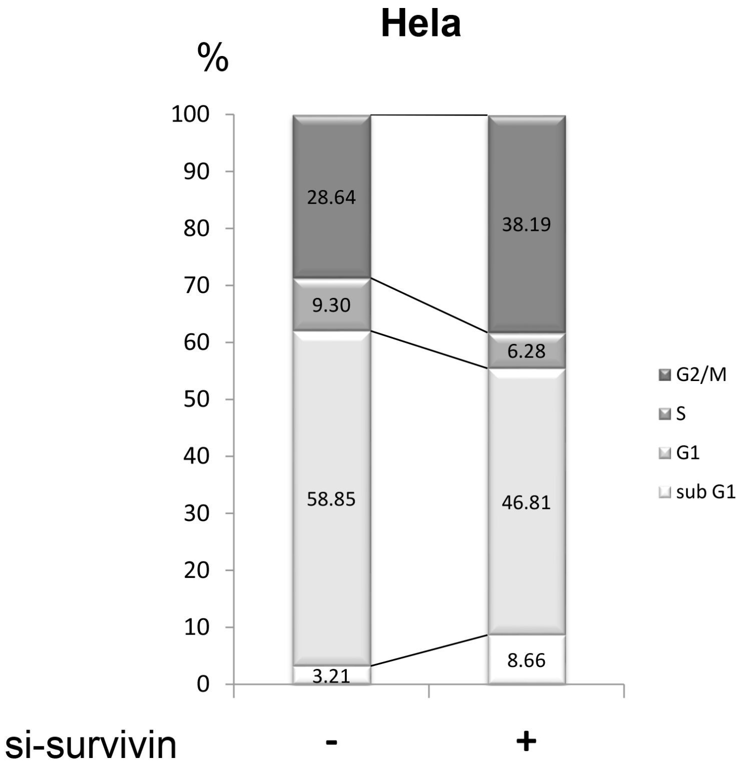

Supplementary Figure 1: Flow cytometric analysis of the cell cycle after survivin knockdown. HeLa cells were transfected with survivin-specific siRNA (si-survivin) for 48 h and then the cell cycle was analyzed. The mean of three independent experiments is shown.

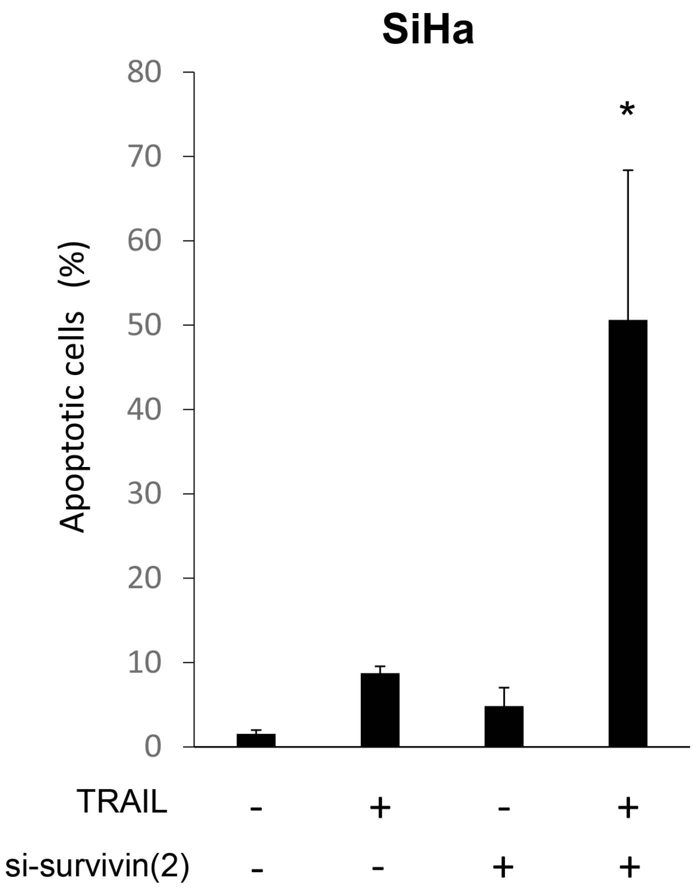

Source	Nparm	DF	SS	F ratio	Prob>F
TRAIL	1	1	2104.90	17.5251	**0.0031**
si-survivin	1	1	1530.70	12.7444	**0.0073**
TRAIL*si-survivin	1	1	1116.12	9.2927	**0.0159**

Supplementary Figure 2: Effects of survivin knockdown on TRAIL-induced apoptosis in SiHa cells. SiHa cells were transfected with control siRNA or survivin-specific siRNA for 48 h, and then treated with TRAIL (100 ng/mL) or not treated for an additional 15 h. The proportion of apoptotic cells was evaluated using annexin-V–fluorescein isothiocyanate (FITC) and propidium iodide (PI) double staining, followed by flow cytometry. The results show the mean of three independent experiments (± SEM). Two-way ANOVA results are provided below. Asterisks (*) indicate that the *p*-value of the interaction effect was < 0.05.

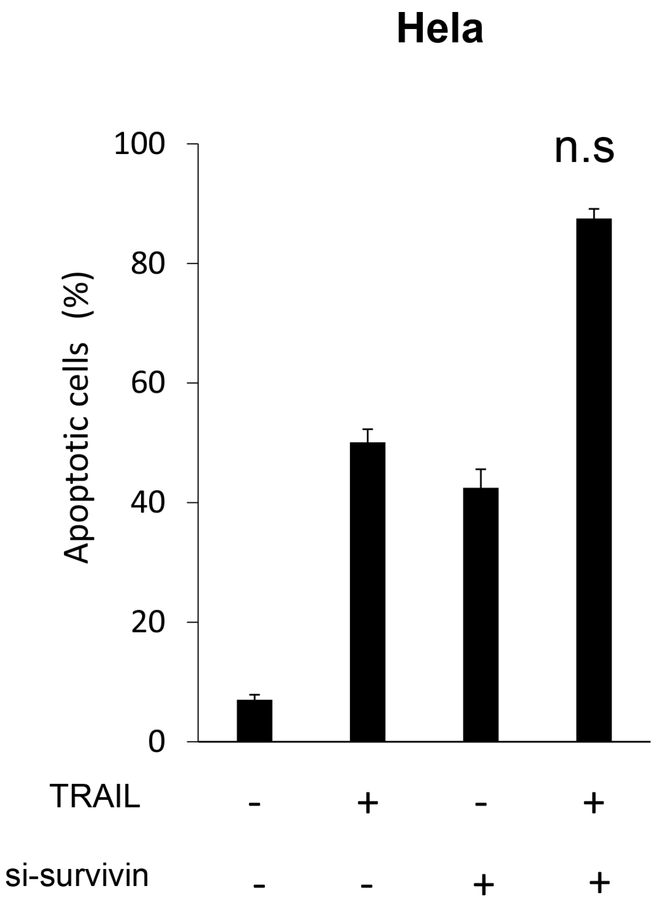

Source	Nparm	DF	SS	F ratio	Prob>F
TRAIL	1	1	6837.80	507.72	**<.0001**
si-survivin	1	1	3217.36	238.89	**<.0001**
TRAIL*si-survivin	1	1	21.74	1.61	**0.2396**

Supplementary Figure 3: Effects of survivin knockdown on TRAIL-induced apoptosis in HeLa cells. HeLa cells were transfected with control siRNA or survivin-specific siRNA for 48 h, and then treated with TRAIL (100 ng/mL) or not treated for an additional 15 h. The proportion of apoptotic cells was evaluated using annexin-V–fluorescein isothiocyanate (FITC) and propidium iodide (PI) double staining, followed by flow cytometry. The results show the mean of three independent experiments (± SEM). Two-way ANOVA results are provided below. n.s.: the interaction effect was not significant.

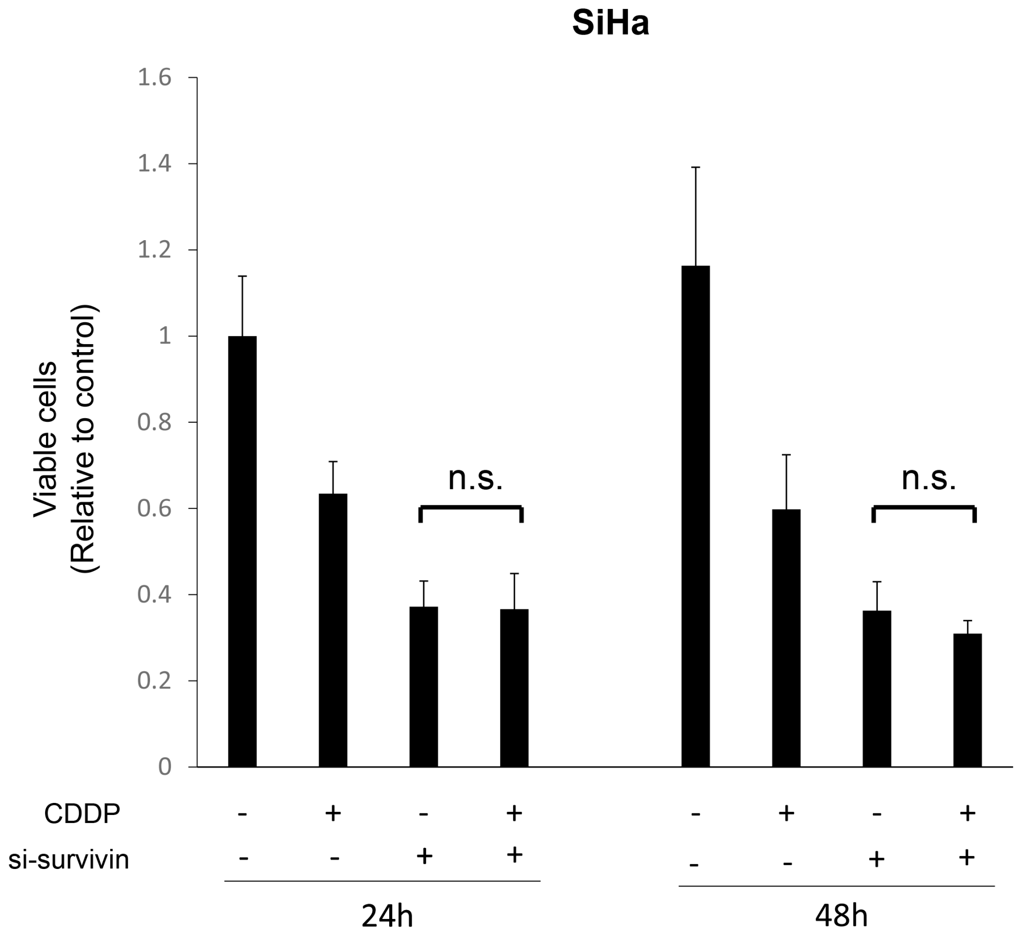

Supplementary Figure 4: Effect of survivin suppression and CDDP combination therapy on cell viability in SiHa cells. SiHa cells were transfected with survivin-specific siRNA (si-survivin) for 48 h and then treated with CDDP (20 μM) for an additional 24 h or 48 h. Adherent cells were counted to assess their viability. The experiment was performed in triplicate. The cell numbers were normalized relative to control cells. Data are provided as mean (±SEM) values. The data were analyzed using Student's *t*-test. n.s.: not significant.
